# The Relationship Between the Big Five Personality Model and Innovation Behavior: A Three-Level Meta-Analysis

**DOI:** 10.3390/bs15091143

**Published:** 2025-08-22

**Authors:** Haidong Zhu, Feihang Jia, Yingxi Zhang, Rui Wang

**Affiliations:** 1 Psychological Application Research Center, Normal College, Shihezi University, Shihezi 832000, China; 2 Education Evaluation Center of Anhui Province, Hefei 230000, China

**Keywords:** innovation behavior, Big Five, meta-analysis, moderating effect, multilevel meta-analysis

## Abstract

Innovation is a driving force in terms of enhancing individual core competencies, fostering social progress, and promoting economic growth. Innovation behavior encompasses all actions involved in the generation of innovative ideas, through to their realization. This study employs a meta-analytic approach to examine the relationship between personality traits and innovation behavior, as well as the potential moderating factors involved. A systematic search of relevant literature in both Chinese and English was conducted, and a total of 91 papers met the inclusion criteria. In total, 399 correlations with a combined sample size of 32,786 were analyzed using the metafor package. The results showed that the four dimensions of the Big Five personality model—agreeableness, extraversion, openness, and conscientiousness—were significantly and positively correlated with innovation behavior. Neuroticism was weakly and negatively associated with innovation behavior. Moderator analyses revealed that the sample type (student vs. employee) and the personality measurement instrument (BFI vs. IPIP) significantly influenced the relationship between openness and innovation behavior. These findings underscore the strong connection between core personality traits and innovation behavior, particularly emphasizing the importance of openness.

## 1. Introduction

As Guilford (1967) noted, the phenomenon of innovation behavior has long been overlooked, yet it has recently gained significant importance. Innovation has gradually become a central measure of talent, and the competitiveness and survival of an organization depend on it. The core source driving innovation behavior is creativity, defined as the ability to generate novel and useful ideas. Divergent thinking is one of the most commonly used indicators for measuring creativity. The advancement of society is largely driven by the development of individual creativity, technological progress, and the shaping of cultural products. Innovation is closely linked to digital transformation (Appio et al., 2021), economic development (Thompson, 2018), and other factors. Numerous studies have examined the factors influencing innovation behavior, separately investigating the effects of factors such as innovation self-efficacy (Akbari et al., 2021), motivation (Gupta, 2020), and knowledge sharing (Shen & Geng, 2020). As the research paradigm of human capital expands to include non-cognitive abilities, personality traits have garnered focused attention in the field of innovation research as a core factor of individual differences. Stable patterns of thoughts, feelings, and behaviors embedded in personality traits (Mowen, 2000) constitute key predictors of innovation behavior. This “trait-behavior” logic (Liu et al., 2020) makes personality an important predictor of individual differences in innovation behavior.

The Big Five personality traits, as a crucial measure of personality, are essential predictors with respect to understanding individual differences in innovation behavior. Numerous studies have examined the impact of personality differences on innovation behavior. Initially, research on personality differences in relation to innovation behavior focused on employee populations; however, as the field evolved, it began to include students. Meanwhile, culture profoundly influences people’s cognition and behavior. Early studies on the Big Five personality model and innovation primarily focused on an individualistic cultural background (Baer & Oldham, 2006). However, research related to collectivist cultural contexts has been increasing steadily (S. Wang & Yi, 2022). As the body of research expands across different cultural backgrounds, it is essential to integrate these studies to explore the relationship between the Big Five personality model and innovative behaviors within the contrasting cultural contexts of individualism and collectivism. Nevertheless, the findings from these studies regarding the Big Five personality model and innovation behavior have been inconsistent.

Based on the above inconsistent research results, Zare and Flinchbaugh comprehensively reviewed the literature from 1980 to 2015 to conduct a meta-analysis of the relationship between the Big Five personality model and innovation behavior. They found significant correlations between extraversion (*r* = 0.27), openness (*r* = 0.46), and conscientiousness (*r* = 0.18) within the Big Five model and innovation behavior. Additionally, they identified sample type as a moderating variable in this relationship. Specifically, the relationship between students’ sense of conscientiousness and innovation behavior was found to be statistically non-significant. (*r* = 0.05), whereas employee conscientiousness exhibited a significant positive correlation with innovation behavior (*r* = 0.23). Neither participant gender nor the source of ratings for innovation behavior moderated the relationship between the two variables (Zare & Flinchbaugh, 2019). The study has several shortcomings: (1) The inconsistency of its findings requires further discussion, as other studies have demonstrated significant positive relationships between agreeableness, extraversion, and innovation behavior (S. Wang & Yi, 2022). (2) The article exclusively included English literature and did not incorporate Chinese empirical studies, possibly overlooking the potential moderating role of cultural context in the relationship between the Big Five personality model and innovative behavior. Therefore, building on Mortaza Zare et al.’s study, this paper conducts a meta-analysis that synthesizes both Chinese and English empirical literature on the Big Five personality model and innovative behavior since 2004. This analysis aims to clarify established academic controversies, better illustrate and explain the relationships between variables, and address the following questions: (1) What is the relationship between the Big Five personality model and innovative behavior. (2) Do the study characteristics (e.g., cultural context, different samples, gender ratio, and type of personality measure) affect the relationship between the Big Five personality model and innovative behavior, i.e., whether they can play a significant moderating role between the two.

## 2. Literature Review

### 2.1. The Big Five Model and Innovation Behavior

As one of the most influential theoretical frameworks in the field of personality psychology, the Big Five personality model has garnered substantial support within academic circles (DeYoung et al., 2007). The Big Five personality model consists of five main factors: agreeableness, extraversion, openness, conscientiousness, and neuroticism.

Agreeableness refers to an individual’s pro-social and collective orientation (John & Srivastava, 1999). Individuals with high agreeableness scores are characterized by a cooperative spirit, generosity, trust, and enthusiasm (DeYoung et al., 2007). Some scholars believe that agreeable individuals tend to prioritize interpersonal harmony and may avoid challenging viewpoints to prevent conflicts (King et al., 1996), which can inhibit the proposal of innovative ideas (Fürst et al., 2016). However, research by Silvia et al. (2011) based on the HEXACO model indicates that there is no significant correlation between agreeableness and creativity; instead, honesty–humility emerges as the key factor in predicting innovation behavior.

Extraversion refers to an individual’s positive attitude toward the social and material world surrounding them (John & Srivastava, 1999). Individuals with high extraversion scores tend to be bold, outgoing, energetic, confident, and adventurous (DeYoung et al., 2007). Numerous studies have demonstrated that extraversion significantly enhances job performance, organizational commitment, and organizational identity. As far as innovative behavior is concerned, extraversion may have an impact through multiple mechanisms. On the one hand, highly extroverted individuals are more likely to actively seek solutions to problems (Joseph & Zhang, 2021), obtain social support, and implement innovative ideas (Yao & Han, 2013). However, there are theoretical differences in the existing research. Some scholars argue that the innovation behavior process fundamentally necessitates deep cognitive processing and introspection (Rank et al., 2004). Additionally, excessive extraversion may result in the over-dispersion of cognitive resources during social activities (Batey et al., 2010). This may account for the phenomenon in which the relationship between extraversion and innovative behavior is not significant in certain studies (J. C. Kaufman & Beghetto, 2013).

Openness to experience is defined by individual differences in the extent to which individuals are open to new experiences, ideas, and wisdom (John & Srivastava, 1999). Individuals high in openness to experience recognize the significance of organizational change (DeYoung et al., 2007) and are consequently more engaged in change-oriented (e.g., employee suggestion, innovation behavior, etc.) proactive behaviors (Chiaburu et al., 2011). It is important to note that the predictive validity of empirical openness is not only evident at the individual level but also serves as a significant predictor of innovation support behavior within national cultural practices in macro-level research (Steel et al., 2012).

Conscientious individuals follow social norms to manage impulsiveness, remain goal-oriented, engage in planning, practice delayed gratification, and adhere to established norms and rules (Roberts et al., 2014). Those with high conscientiousness scores are typically characterized by their reliability, practicality, and diligence (DeYoung et al., 2007). This diligence can foster innovation through persistence (King et al., 1996). When confronted with challenges, the sense of responsibility among highly conscientious individuals is significantly heightened. This internal motivation compels them to actively seek out problem-solving solutions, thereby increasing the likelihood of creative problem-solving (Xue et al., 2020). Furthermore, research by Poropat (2009) indicates that cognitive and behavioral traits associated with conscientiousness, such as self-discipline and deliberation, can effectively mitigate deficiencies in certain areas of individual ability.

Neuroticism refers to an individual’s tendency to experience negative emotional states (John & Srivastava, 1999). Those with higher neuroticism scores are typically characterized by traits such as nervousness, irritability, restlessness, and emotional instability (DeYoung et al., 2007). Conversely, individuals with lower neuroticism scores, indicating greater emotional stability, tend to exhibit calmness, relaxation, and dispassion. Most studies have demonstrated a negative correlation between neuroticism and innovation (Raja & Johns, 2010). However, some research indicates no correlation between neuroticism and individual innovation (King et al., 1996). Additionally, certain studies suggest that moderate levels of anxiety can serve as a motivator for creative problem-solving (Perkins et al., 2015).

### 2.2. Moderators

By reviewing previous studies and examining related literature, this study identified four moderating variables: cultural context, sample type, gender differences, and types of personality measurement. The following section discusses the main factors related to the Big Five personality model and innovation behavior.

#### 2.2.1. Cultural Context

According to culture-matching theory, national culture influences individual behaviors and outcomes (J. Wang et al., 2022). The level of individualism within a society reflects the importance that individuals place on self-awareness and personal goals. Conversely, collectivism represents the extent to which individuals value interpersonal cohesion and prioritize the interests of the collective over those of the individual (Schwartz & Bilsky, 1990). While it is widely acknowledged that individualism and collectivism profoundly impact innovation behavior, existing empirical research presents conflicting results. In individualism, individuals are more inclined to pursue their own goals and achievements; thus, such cultures tend to promote risk taking (Bradley et al., 2013) and are more likely to generate creative ideas and foster innovation behavior (Desmarchelier & Fang, 2016). However, some empirical studies indicate that patriotism and nationalism within collectivist cultures can positively influence innovation behavior (M. Z. Taylor & Wilson, 2012). In a study by Zheng et al. (2023) involving Chinese participants, it was found that region significantly moderated the relationship between openness, extraversion, and subjective well-being within the Big Five personality model. Specifically, individuals from Hong Kong, Macao, and Taiwan, influenced by Western culture, exhibited a significantly stronger correlation between openness, extraversion, and well-being compared to their counterparts in mainland China. These findings suggest that personality and creative thinking vary across different cultures. Therefore, this paper analyzes the cultural perspectives of collectivism and individualism as moderating variables, drawing on Hofstede’s (2001) cultural theory.

#### 2.2.2. Sample Type

Innovation behavior is not confined to the workplace; it is equally vital within the student population. Student innovation behavior has emerged as a prominent research topic in both education and psychology. Education fosters students’ capacity for innovation behavior and scientific thinking, transforming them into high-quality human resources and enhancing the creativity and quality of the workforce. These human resources play a crucial role in driving innovation behavior and bolstering the innovation behavior dynamics of companies. Scholars have raised concerns that employee samples from various industries may not be generalizable to other work environments (Hubbard & Vetter, 1996). When comparing student samples to other types of samples, research has shown that students exhibit greater openness (*r* = 0.24) and artistic creativity than other samples (*r* = 0.13) (S. B. Kaufman et al., 2016). Consequently, even if the independent and dependent variables remain constant in a study, variations in the study population can significantly influence the results.

#### 2.2.3. Gender Differences

Gender differences in personality are considerable. Generally, men tend to score lower on agreeableness and neuroticism while scoring higher on intellectual curiosity and unconventionality (Lee & Ashton, 2020). Additionally, research indicates that gender influences the type of tasks in innovation behavior, with males exceling in problem solving and females outperforming in divergent thinking and creative work (C. L. Taylor et al., 2024). Furthermore, there is a notable gender disparity in the relationship between the Big Five personality traits and workers’ salaries. Agreeableness and emotional stability (neuroticism) are more significant for female workers, whereas conscientiousness has a greater impact on male workers (Le & Hu, 2017).

#### 2.2.4. Personality Measurement Types

Based on previous studies (Anglim et al., 2022; Grajzel et al., 2023), the present study includes personality type measures as a moderating variable. This paper considers four commonly used Big Five personality questionnaires, while different questionnaires used in combination are classified under the “Others” category, which contains the following four measures: the NEO Personality Inventory, the International Personality Item PoolorMini-IPIP (IPIP), the Ten-Item Personality Inventory (TIPI), and the Big Five Inventory (BFI). The BFI, developed by John, defines openness as encompassing the breadth, depth, originality, and complexity of an individual’s psychological and experiential life. It includes dimensions such as originality and artistic experience, both of which are related to innovation behavior (John & Srivastava, 1999). The IPIP questionnaire characterizes the openness trait as intellectual, featuring items primarily associated with intellectual orientation (e.g., imagination, curiosity) while excluding several aspects of the broader openness construct, such as artistic interests and liberalism (Mammadov, 2022). These questionnaires exhibit both subtle and significant differences.

## 3. Method

This study was conducted following the guidelines of the Preferred Reporting Items for Systematic Review and Meta-Analysis (PRISMA) (Page et al., 2021) and was preregistered under the Open Science Framework (OSF) (registration number: 10.17605/OSF.IO/9H567).

### 3.1. Literature Search

**Figure 1:**
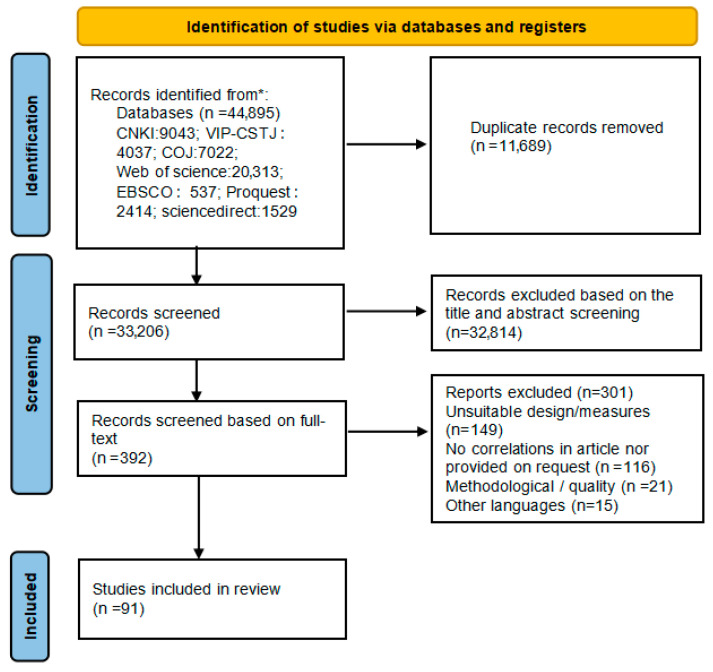
PRISMA chart. *Consider, if feasible to do so, reporting the number of records identified from each database or register searched (rather than the total number across all databases/registers).

The search was conducted in both English and Chinese, with the search period set for December 2024. We used “subject” as the search term and searched relevant Chinese and English databases by modifying search expressions and employing Boolean logical operators for connections. The Chinese search utilized the China National Knowledge Infrastructure (CNKI), Wanfang (China Online Journals, COJ), and VIP (VIP-CSTJ) databases. The search terms related to the Big Five personality traits included “big five personality”, “personality”, “neuroticism”, “conscientiousness”, “extraversion”, “pleasantness”, “rigor”, “openness (to experience)”, “dutifulness”, and “extraversion”; search terms for innovation behavior were “innovation”, “innovative behavior”, “innovative performance”, “creativity”, “idea generation”, “idea implementation”, and “idea promotion”. The keywords were paired and searched. The English searches were conducted in foreign-language databases such as Web of Science, Springer Link, ProQuest, and Science Direct. The search terms related to the Big Five personality search terms were personality, Big Five, NEO, Big Five Inventory, BFI, Five Factor Model, neuroticism, conscientiousness, openness, extraversion, and agreeableness; search terms for innovative behavior were innovat*, creativity, innovative behavi*, creative behavi*, innovative performance, creative performance, idea generation, idea implementation, and idea promotion. Keywords were searched in pairs. During the search process, meta-analysis papers and review articles related to the Big Five personality model and innovation behavior were compared to ensure that no relevant literature was overlooked. Additionally, literature on related topics that may have been missed was incorporated by reviewing the references of the downloaded papers. A flowchart of the literature screening process is shown in Figure 1.

### 3.2. Eligibility Criteria

Combining the fundamentals of meta-analytic research methods with the research topic, this study establishes the following inclusion and exclusion criteria: (1) The study investigates the relationship between the Big Five personality model and innovation behavior, and the research data must be complete. Purely theoretical works, literature reviews, and meta-analytic articles are excluded. (2) The study must be empirical and include information about the sample size, correlation coefficients, or other data indicators that can be converted into correlation coefficients, such as F values, t values, chi-square values, and regression coefficients or path coefficients. If a study employed structural equation modeling and did not provide a correlation coefficient between the two variables, it was excluded. The samples must be independent across studies; if the same sample was used in two different studies with identical measurements, only one study was included. If a dissertation was published in an academic journal, the published journal article took precedence. (3) The sample size in the study must be clearly defined. The Big Five personality model is divided into five dimensions, and the samples within each dimension must be independent. If the same sample appears in multiple studies, it is included only once, following the principle of publication time priority. If a single study contains multiple research samples from various sub-studies, each research sample is coded independently. (4) Literature must be published in either Chinese or English. Cross-level studies that primarily explore the effects of leadership personality traits on employee innovation behavior were excluded, as they deviate from the original intent of this study, which is to conduct a meta-analysis of the relationship between personality and innovation behavior at the same level. (5) Publicly available literature was searched from January 2004 to December 2024, focusing on literature from the last 20 years to emphasize contemporary research practices. (6) Due to the variety in methods of measuring innovation behavior, only self-reported measures are included in this paper. Based on the aforementioned literature screening principles, this study ultimately identified valid literature published between 2004 and 2024. In total, we analyzed 68 effect sizes for agreeableness, 73 for extraversion and neuroticism, 102 for openness, and 83 for conscientiousness, drawn from 91 studies with a total sample of 32,786 participants.

### 3.3. Data Extraction

After identifying valid studies for inclusion in the meta-analysis, the following data were extracted from each independent sample for coding in this study: basic information, such as the title of the paper, the year of publication, the authors, and the sample size, as well as research information, including the correlation coefficients for each dimension of the Big Five personality model and innovation behaviors (or the F value, t value, and chi-square value reported in experimental papers). Additionally, moderating variables such as cultural context, sample type, gender (female ratio), and the type of personality measure were coded. Among them, in the coding of cultural background, we utilized Hofstede’s Individualism Index to measure the level of individualism. This index is scored on a 100-point scale, where a higher score indicates a greater degree of individualism, while a lower score reflects a higher level of collectivism (Hofstede, 2001). Although this index has faced criticism for overlooking cultural differences within countries and for being overly simplistic, it has been extensively replicated and widely applied in cross-cultural studies.

### 3.4. Data Analytic Approach

For all effect sizes of the relationship between the Big Five personality model and innovation behavior, 379 of them were reported as correlation coefficients and 20 as linear regression coefficients (β). All linear regression coefficients (β) were transformed to r (Roth et al., 2018).

Meta-analytic correlations were estimated using a random-effects model with the metafor package in R4.4.3 (Viechtbauer, 2010). First, a three-level meta-analysis was conducted (Assink & Wibbelink, 2016) to estimate the average effect size of the Big Five personality model and innovation behavior and assess the impact of potential moderators. Traditional meta-analysis cannot overcome the challenge of multiple independent effect sizes being included in a single study. Even averaging these multiple effects will lead to information loss and inaccurate estimation (Van Den Noortgate & Onghena, 2003). However, all the effect sizes of a study can be included in the three-level meta-analysis by dividing the sources of variance into (a) sampling variance, (b) within-study variance, and (c) between-study variance.

This study employed Egger–MLMA regression to assess publication bias. When effect sizes are not independent of one another, Egger–MLMA regression is more effective in controlling for Type I error compared to the traditional Egger test (Rodgers & Pustejovsky, 2021). If significant publication bias is present, the Egger–MLMA test will yield significant results. In cases of significant publication bias, further testing and correction are necessary using the trim-and-fill method (Duval & Tweedie, 2000). Publication bias is indicated if Ro^+^ > 3 and Lo^+^ > 2 (Fernández-Castilla et al., 2021).

## 4. Results

### 4.1. Heterogeneity Analysis

A heterogeneity test was conducted for all effect sizes. The results indicated high heterogeneity across all overall models (QE(398) = 17,616.488, *p* < 0.001, I^2^ = 97.39%). Further analysis revealed significant within-study variance (LRT = 12,122.98, *p* < 0.001) and between-study variance (LRT = 7.52, *p* < 0.001). Of the total sources of variance, sampling variance, within-study variance, and between-study variance accounted for 2.62%, 89.49%, and 7.89%, respectively. Therefore, it is essential to further investigate the moderating role of variables between the Big Five personality model and innovation behavior.

### 4.2. Publication Bias

In this study, Egger–MLMA regression and trim-and-fill analysis were employed to assess publication bias. The results of Egger–MLMA regression for agreeableness, openness, and neuroticism were not significant (agreeableness, *p* = 0.996; openness, *p* = 0.236; neuroticism, *p* = 0.267). In contrast, the regression results for extraversion and conscientiousness were significant (extraversion, *p* < 0.05; conscientiousness, *p* < 0.05); however, the trim-and-fill analysis indicated no publication bias in these two dimensions (Ro^+^ = 0 < 3 and Lo^+^ = 0 < 2). This suggests that there is no significant publication bias in the current model.

### 4.3. Overall Effect Size

The results of the main test of the effect the Big Five personality dimensions on innovation behavior are presented in Table 1. Given that innovative behavior, divergent thinking, creativity, and innovation performance are inter-related in meaning, this study employs the existing variable modular operation method to enhance the overall degree of effect aggregation (Viswesvaran & Ones, 1995). Openness to experience (*r* = 0.406), conscientiousness (*r* = 0.292), extraversion (*r* = 0.351), and agreeableness (*r* = 0.116) were all significantly and positively correlated with innovation behavior. Gignac and Szodorai (2016) proposed that correlation coefficients of *r* = 0.1, *r* = 0.2, and *r* = 0.3 be interpreted as low, medium, and strong correlations, respectively. Therefore, openness and extraversion are strongly correlated with innovation, conscientiousness shows a moderate correlation, and agreeableness exhibits a low correlation with innovation behavior, while neuroticism (*r* = −0.083) exhibits a weakly negative correlation with innovation behavior.

### 4.4. Moderator Analysis

The results of the heterogeneity test indicated a significant degree of heterogeneity, necessitating an exploration of its sources, specifically the moderating variables that may influence the relationships between the Big Five personality dimensions and innovation behavior. In this study, tests for moderating effects were conducted on categorical variables, including sample type and personality measure type, as well as on continuous variables such as cultural context and gender differences (female ratio).

We employed the meta-regression method to examine the moderating effect of the cultural context on the Big Five personality model. As shown in Table 2, cultural context did not have a significant moderating effect on the relationship between the Big Five personality dimensions and innovation behavior. This indicates that the connection between the Big Five personality model and innovation behavior is not influenced by cultural context. Agreeableness, extraversion, openness, and conscientiousness were all significantly and positively correlated with innovation behavior, whereas neuroticism exhibited a non-significant negative correlation with innovation behavior.

As shown in Table 3, sample type significantly moderated the relationship between openness and innovation behavior. The correlation coefficients between openness and innovation behavior were notably higher in the student sample than in the employee sample. The remaining four dimensions of the Big Five personality model—agreeableness, extraversion, conscientiousness, and neuroticism—did not exhibit a significant moderating effect on innovation behavior. The employee sample demonstrated significant positive correlations for agreeableness, extraversion, and conscientiousness, along with a non-significant negative correlation for neuroticism. The student sample revealed significant positive correlations for agreeableness, extraversion, and conscientiousness, as well as a non-significant negative correlation for neuroticism.

We employed the meta-regression method to examine the moderating effect of the gender ratio (female ratio) on the Big Five personality model. As shown in Table 4, gender does not have a significant moderating effect on the relationship between the Big Five personality dimensions and innovation behavior. This indicates that the connection between the Big Five personality dimensions and innovation behavior is not influenced by gender. Females exhibited a significant positive correlation with agreeableness, extraversion, openness, and conscientiousness while demonstrating a negative correlation with neuroticism.

As shown in Table 5, the method of measuring personality significantly moderated the relationship between openness and innovation behavior. The correlation coefficient between openness and innovation behavior was notably higher when using the BFI questionnaire compared to the IPIP questionnaire. No significant moderation was observed between the remaining four dimensions of the Big Five personality model—agreeableness, extraversion, conscientiousness, and neuroticism—and innovation behavior. Regardless of the instrument employed, agreeableness exhibited a positive but not statistically significant association with innovation behavior. Extraversion demonstrated a positive correlation with innovation behavior and was significantly associated with innovation behavior across the various measures. When utilizing the TIPI questionnaire, no significant correlation was found between conscientiousness and innovation behavior. However, neuroticism was significantly and negatively correlated with innovation behavior when assessed using the BFI questionnaire.

## 5. Discussion

This study integrated 91 research articles using a three-level meta-analysis approach to provide a comprehensive analysis of the relationship between the Big Five personality model and innovation behavior. The findings revealed that agreeableness, extraversion, openness, and conscientiousness were significantly positively correlated with innovation behavior, while neuroticism exhibited a weakly negative correlation. Specifically, openness and extraversion demonstrated the strongest associations with innovation behavior, followed by agreeableness and conscientiousness, which showed slightly weaker relationships. In contrast, neuroticism had the weakest negative correlation with innovation behavior. Among the moderators identified in the study, sample type and personality measurement were found to influence the relationship between openness and innovation behavior. This finding is not entirely consistent with the conclusions drawn by Zare and Flinchbaugh (2019) in their meta-analysis of English literature from 1980 to 2015. One possible explanation is that as related research has progressed, the characteristics of the subjects in studies examining the relationship between the Big Five personality dimensions and innovation have become more diverse. This includes a broader range of geographical regions, a wider variety of industries, and more comprehensive demographic features. Consequently, the overall sample data has become more representative and balanced, making the conclusions of the meta-analysis more reflective of the actual situation.

Many studies have concluded that openness has the most consistent effect on innovation behavior, as it is considered a personality trait closely linked to intelligence, creativity, aesthetics, and other factors (Anglim et al., 2022). The intellectually related aspect of openness can promote innovation behavior through knowledge accumulation (crystallized intelligence), while the aesthetically related aspect stimulates metaphorical thinking, which enhances innovation behavior in design (Sanchez-Ruiz et al., 2013). Individuals high in extraversion are often bold, confident, and socially adept; certain features of extraversion can indirectly influence innovation behavior by fostering positive emotions, such as happiness and euphoria (Baas et al., 2008). Agreeableness and conscientiousness, two personality traits with weaker relationships with innovation behavior, may still influence innovation behavior through other factors.

The results indicated that cultural context did not significantly moderate the relationship between the Big Five personality dimensions and innovation behavior. Consistent with Katalin’s findings, cultural differences did not play a moderating role in the relationship between personality and divergent thinking, which is one of the most commonly used indicators of creative potential (Grajzel et al., 2023). Although it is widely believed that individualistic and collectivistic cultures can differ in personality traits, research has shown that the Big Five personality structure is stable across cultures (McCrae & Terracciano, 2005). Furthermore, with the advancement of economic globalization and the rise of multinational enterprises, the core elements of innovation behavior are increasingly recognized as universal. Innovation behaviors are often directed toward common goals, such as profit maximization, which may diminish the moderating influence of cultural context on the relationship between personality and innovation behavior. Globalization also contributes to the convergence of innovation behavior standards (Chiu & Kwan, 2010). On the other hand, the sample type in this meta-analysis is geographically concentrated, with 58 out of 91 samples originating from China. This limitation may hinder the study’s ability to adequately reflect cultural differences. Consequently, these factors may have contributed to the less significant moderating role of cultural context in the relationship between personality and innovation behavior.

In the moderated effects analysis of sample type, it was found that the correlation coefficient between students’ openness and innovation behavior was significantly higher than that between employees’ openness and innovation behavior. Students are in a school environment that typically encourages exploration of subjects and the cultivation of divergent thinking. They can generate innovative ideas through knowledge sharing with teachers or classmates, benefiting from a lower cost of innovation behavior and a more inclusive atmosphere. Consequently, students with high levels of openness are adept at innovating by utilizing the resources available to them. According to the ego depletion theory, each individual’s psychological resources are limited. Engaging in volitional activities depletes these resources, which can reduce the availability of psychological resources for subsequent behaviors (Muraven et al., 1998). Employee innovation behavior must consider various factors, including workplace elements such as budget, time, and organizational culture (Morrison, 2011), as well as family dynamics. When employees expend more resources at work and at home, they may invest less effort in innovation behavior. Additionally, competitive interpersonal relationships in the workplace and limited communication among coworkers can weaken the connection between employees’ openness and innovation behavior. Second, there may be age differences between students and employees, with the student population typically ranging from 18 to 25 years old, while the employee population encompasses a broader age range. Age is highly correlated with sample type. Openness is generally higher in early adulthood and tends to stabilize or decline with age (Roberts et al., 2006). The aging process is associated with the maturation of regulatory mechanisms, which also influences the relationship between openness and innovation behavior. The remaining four personality traits were not moderated by sample type, indicating that they were equally significant in influencing innovation behavior across the different sample groups of students and employees.

Consistent with previous findings, the moderating effect of gender on the relationship between the Big Five personality model and innovation behavior was not significant. Modernization theory posits that as economic structures transition from agricultural to industrial and, ultimately, post-industrial societies, citizens become increasingly receptive to post-materialist values, such as gender equality (Stockemer & Sundström, 2016). This evolution is accompanied by a weakening of traditional gender-role norms that restrict innovative behavior. The expansion of education and the rise of female empowerment reduce the disparities in access to resources between men and women. Consequently, personality traits, rather than gender, emerge as the primary predictors of innovation behavior. Post-industrial societies place greater emphasis on competence-based roles rather than a gendered division of labor, and the cognitive flexibility exhibited by both men and women with high levels of openness can foster innovation behavior. Additionally, meta-analyses indicate minimal gender differences in innovation behavior competence (C. L. Taylor et al., 2024). These factors may explain the insignificant moderating role of gender in the relationship between personality and innovation behavior.

The type of personality measure moderated the relationship between two factors of openness and innovation behavior. The results indicated that the correlation coefficient between openness and innovation behavior was as high as 0.548 in the BFI questionnaire but only 0.287 in the IPIP questionnaire. This discrepancy may stem from the BFI questionnaire’s emphasis on behavioral representations, where the dimensions of openness (e.g., imagination and willingness to try new things) are more closely aligned with the direct behavioral drivers of innovation behavior (Rammstedt & John, 2007). Furthermore, the BFI’s openness contains the fantasy and aesthetics sub-dimensions, both of which are associated with divergent thinking and cross-domain associations, thereby facilitating the innovation behavior process. In contrast, the IPIP questionnaire exhibits the lowest correlation coefficient for openness, likely because its questions are designed to be streamlined, enhancing utility but also limiting the portrayal of its complex qualities. The IPIP questionnaire tends to be more biased toward cognitive tendencies. The openness dimension primarily assesses individuals’ interest in abstract concepts and lacks direct measurement of certain elements of innovation behavior (e.g., risk-taking, creative output, etc.) (Donnellan et al., 2006). In addition, the correlations between different measurement tools vary across personality dimensions. For the openness dimension, the correlation coefficient for the BFI (*r* = 0.548) is higher than that for the NEO (*r* = 0.410). This discrepancy may be related to the reliability and validity of the measurement instruments. Some studies have indicated that, compared to other questionnaires, the NEO-PI-R demonstrates higher reliability and more consistent results, whereas the BFI exhibits lower reliability (John & Srivastava, 1999). Therefore, differences in the reliability and validity of these measurement tools may contribute to variations observed in correlation analyses. The relationship between the remaining four personality traits and innovation behavior was not significantly moderated by the type of personality measure, suggesting that the connection between these traits and innovation behavior is less influenced by the measurement method.

## 6. Limitations and Future Research

This study provides a comprehensive assessment of the relationship between the Big Five personality model and innovation behavior; however, several limitations must be considered. First, because measures of innovation behavior are categorized into various types—such as self-reports, behavioral tasks, and objective outcome indicators—and because each type includes numerous questionnaires developed by different scholars, this paper only includes self-reported measures in our analysis. This limitation may lead to issues such as social desirability bias or misalignment between subjective and objective assessments, which do not accurately reflect individuals’ true innovative abilities. Additionally, in cross-cultural studies, individual responses from collectivist cultures may be more conservative, further exacerbating measurement bias (Heine, 2001). Future studies could categorize measures of innovation behavior in greater detail, analyzing and comparing behavioral tasks and self-reported studies separately to yield more comprehensive results. Second, this study only examines the effects of the five dimensions of the Big Five personality model on innovation behavior. Understanding the impact of each facet of these traits on innovation behavior could provide a clearer explanation of the relationship between personality and innovation behavior. Future research could focus more on specific structural facets and offer diverse analytical perspectives to elucidate the connection between personality and innovation behavior.

## 7. Conclusions

This study employed a three-level meta-analysis to systematically examine the relationship between the Big Five personality model and innovation behavior. The following conclusions were drawn: First, in the comprehensive relationship, agreeableness, extraversion, openness, and conscientiousness were found to be significantly positively correlated with innovation behavior, while neuroticism exhibited a weakly negative correlation with innovation behavior. Among these traits, openness emerged as the strongest predictor. Second, in terms of moderating factors, cultural context and gender did not significantly influence the relationship between the Big Five personality model and innovation behavior. However, sample type and the method of personality measurement did moderate the relationship, specifically within the openness dimension. Notably, the effect sizes for openness and innovation behavior were significantly higher among students compared to employees. Additionally, when the BFI questionnaire was utilized, the effect sizes for openness and innovation behavior were significantly greater than those obtained from other questionnaires.

## Figures and Tables

**Table 1:** Meta-analytic correlations between the Big Five personality traits and innovation behavior.

Trait	*k*	#es	N	*r*	*t*	95%CI	%Var.at Level 1	%Var.at Level 2	%Var.at Level 3
Agreeableness	48	68	18,286	0.116 *	2.459	[0.022, 0.211]	2.096	4.176	93.728
Extraversion	53	73	19,039	0.351 ***	10.700	[0.286, 0.417]	4.305	15.006	80.688
Openness	77	102	28,875	0.406 ***	14.685	[0.31, 0.461]	4.41	13.956	81.635
Conscientiousness	61	83	22,253	0.292 ***	8.566	[0.224, 0.36]	3.566	9.409	87.024
Neuroticism	53	73	19,143	−0.083	−2.066	[−0.163, −0.003]	2.862	9.524	87.613

Note: *k*, number of studies; #es, number of effect sizes; N, total sample size; *t*, *t* test value for the difference between mean effect size and 0; *r*, transformed effect size (*r*); CI, confidence interval; %Var, percentage of variance that is distributed at one of the three levels of the meta-analytic model (Level 1, sample variance; Level 2, variance between effect sizes from the same study; Level 3, variance between studies). *, *p* < 0.05; ***, *p* < 0.001. The same below.

**Table 2:** Summary of analyses of moderation effect of cultural context.

	Moderator	Q_E_(df)	F(df1, df2)	*r*	SE	95%CI	*t*
Agreeableness	Cultural context (Individualism)	Q_E_(57) = 1146.122 ***	F(1, 57) = 0.885 ***	0.156	0.036	[0.083, 0.228]	4.312
Extraversion	Cultural context (Individualism)	Q_E_(62) = 123.391 ***	F(1, 62) = 0.275 ***	0.368	0.035	[0.298, 0.439]	10.465
Openness	Cultural context (Individualism)	Q_E_(88) = 1604.224 ***	F(1, 88) = 0.000 ***	0.398	0.030	[0.339, 0.457]	13.382
Conscientiousness	Cultural context (Individualism)	Q_E_(32) = 474.630 ***	F(1, 70) = 2.003 ***	0.309	0.047	[0.237, 0.382]	8.507
Neuroticism	Cultural context (Individualism)	Q_E_(62) = 1639.618 ***	F(1, 62) = 0.458	−0.082	0.044	[−0.170, 0.006]	−1.867

Note: ***, *p* < 0.001.

**Table 3:** Summary of analyses of moderation effect of sample type.

	Moderator	Q_E_(df)	F(df1, df2)	*r*	SE	95%CI	*t*
Agreeableness	Sample type	Q_E_(55) = 941.155 ***	F(1, 55) = 0.819 **				
	Employee			0.217	0.069	[−0.235, 0.354]	3.161
	Students			0.144	0.042	[0.059, 0.229]	3.385
Extraversion	Sample type	Q_E_(60) = 1025.024 ***	F(1, 60) = 0.103 ***				
	Employee			0.380	0.062	[0.257, 0.504]	6.173
	Students			0.356	0.043	[0.270, 0.443]	8.224
Openness	Sample type	Q_E_(89) = 1580.689 ***	F(1, 89) = 6.527 ***				
	Employee			0.340	0.040	[0.261, 0.418]	8.575
	Students			0.480	0.038	[0.404, 0.557]	12.538
Conscientiousness	Sample type	Q_E_(70) = 1530.131 ***	F(1, 70) = 0.226 ***				
	Employee			0.326	0.056	[0.215, 0.437]	5.849
	Students			0.291	0.048	[0.196, 0.386]	6.087
Neuroticism	Sample type	Q_E_(60) = 1543.681 ***	F(1, 60) = 0.713				
	Employee			−0.033	0.076	[−0.184, 0.119]	−0.431
	Students			−0.111	0.053	[−0.217, −0.005]	−2.086

Note: **, *p* < 0.01; ***, *p* < 0.001.

**Table 4:** Summary of analyses of moderation effect of gender distribution.

	Moderator	Q_E_(df)	F(df1, df2)	*r*	SE	95%CI	*t*
Agreeableness	Gender (Female %)	Q_E_(59) = 4858.455 ***	F(1, 59) = 3.374 *	0.123	0.050	[0.023, 0.224]	2.448
Extraversion	Gender (Female %)	Q_E_(64) = 1159.998 ***	F(1, 64) = 2.867 ***	0.333	0.032	[0.269, 0.387]	10.422
Openness	Gender (Female %)	Q_E_(91) = 1888.765 ***	F(1, 91) = 0.488 ***	0.414	0.030	[0.355, 0.473]	13.923
Conscientiousness	Gender (Female %)	Q_E_(72) = 1543.624 ***	F(1, 72) = 0.039 ***	0.296	0.035	[0.227, 0.365]	8.517
Neuroticism	Gender (Female %)	Q_E_(63) = 1702.415 ***	F(1, 63) = 0.352 *	−0.095	0.045	[−0.184, −0.006]	−2.141

Note: *, *p* < 0.05; ***, *p* < 0.001.

**Table 5:** Summary of analyses of moderation effect of personality measurement.

	Moderator	Q_E_(df)	F(df1, df2)	*r*	SE	95%CI	*t*
Agreeableness	Personality Measurement	Q_E_(63) = 4999.632 ***	F(1, 63) = 0.264				
	NEO			0.128	0.074	[−0.019, 0.275]	1.737
	IPIP			0.026	0.108	[−0.190, 0.242]	0.241
	TIPI			0.166	0.155	[−0.144, 0.475]	1.071
	BFI			0.17	0.115	[−0.060, 0.401]	1.479
	Others			0.1	0.171	[−0.243, 0.442]	0.583
Extraversion	Personality Measurement	Q_E_(70) = 1530.131 ***	F(1, 67) = 0.749 ***				
	NEO			0.352		[0.252, 0.452]	7.014
	IPIP			0.263		[0.118 ,0.407]	3.633
	TIPI			0.282		[0.072, 0.492]	2.68
	BFI			0.435		[0.279, 0.591]	5.571
	Others			0.353		[0.164, 0.541]	3.725
Openness	Personality Measurement	Q_E_(95) = 1715.517 ***	F(4, 95) = 2.712 ***				
	NEO			0.410	0.043	[0.325, 0.496]	9.532
	IPIP			0.287	0.056	[0.176, 0.398]	5.142
	TIPI			0.463	0.074	[0.316, 0.610]	6.253
	BFI			0.548	0.065	[0.419, 0.678]	8.417
	Others			0.307	0.104	[0.101, 0.512]	2.961
Conscientiousness	Personality Measurement	Q_E_(60) = 1025.024 ***	F(4, 77) = 0.547 **				
	NEO			0.344		[0.235, 0.454]	6.256
	IPIP			0.257		[0.124, 0.389]	3.872
	TIPI			0.223		[−0.017, 0.464]	1.85
	BFI			0.270		[0.100, 0.439]	3.171
	Others			0.197		[−0.040, 0.434]	1.656
Neuroticism	Personality Measurement	Q_E_(67) = 1398.499 ***	F(4, 2) = 0.692				
	NEO			−0.028	0.062	[−0.153, 0.097]	−0.449
	IPIP			−0.09	0.083	[−0.255, 0.076]	−1.08
	TIPI			−0.037	0.131	[−0.287, 0.244]	−0.28
	BFI			−0.221 *	0.103	[−0.426, −0.015]	−2.145
	Others			−0.04	0.129	[−0.297, 0.218]	−0.307

Note: *, *p* < 0.05; **, *p* < 0.01; ***, *p* < 0.001.

## Data Availability

The data that support the findings of this study are openly available in the Supplementary Materials (Table S1 and File S1).

## Supplementary Materials

The following supporting information can be downloaded at: https://www.mdpi.com/article/10.3390/bs15091143/s1, Table S1: Coding results; File S1: Code in Meta-analysis.
