# Supplementary material for: The Relationship Between the Big Five Personality Model and Innovation Behavior: A Three-Level Meta-Analysis
Source: Behav Sci (Basel). 2025 Aug 22;15(9):1143. doi: 10.3390/bs15091143 (PMC12466565; doi:10.3390/bs15091143)
Supplement: Supplementary file 1 [file behavsci-15-01143-s001.zip › R code.pdf]

Code in Meta-analysis

```
> # step 1: Install metafor package ####
# install.packages("metafor")
> library(metafor)

> # step 2: Reading data ####
> # reading data from CSV to R
> meta.csv <- "C:/Users/86182/Desktop/2.24Meta_data.csv"
> Q0224meta <- read.table(file = meta.csv, header = TRUE, nrows = 399, sep = ",")
Q0224meta <- read.table(file = meta.csv, header = TRUE, nrows = 83, sep = ",",fileEncoding = "GB18030")

> #showing all data
> View(Q0224meta)
> dataset <- Q0224meta

> # step 3: calculating effect size
> # The first step is to transform r to Z and calculate the corresponding sample variances.
> #metafor： the function that calculates the effect size, and slab denotes the optional vector with labels for the studies.
> dataset <-escalc(measure="ZCOR", ri=r, ni=N, data=dataset)
> dataset
```

overall associations

```
> # step 4: Estimating the overall effect by fitting an intercept-only model ####
> # significant overall effect between A and B
> overall <- rma.mv (yi, vi, random = list (~ 1 | EID, ~ 1 | SID), tdist = TRUE, data= dataset)
> summary (overall, digits=3)
```

Forest plot

```
> ## Forest plot for multilevel meta-analysis
> res <- rma(yi, vi, data=dataset, slab=Studyname)
> res

> png("Forest.png" , width = 2000, height = 14000,units = "px", res = 300)
>
> k=nrow(dataset)
> forest(res, xlim  = c(-3, 3 ),
+       atransf =transf.ztor,
+       at =transf.rtoz(c(-.9,0,.9)),
+       digits =c(2,1),
+       cex =0.8,
+       ylim = c(0, k+3))
> dev.off()
```

# within-study variance (non-significant)

```
> # step 5: Estimating the variance of the overall effect size ####
> # step 5.1: build a two-level model without within-study variance
```

```
> modelnovar2 <- rma.mv (yi, vi, random = list (~ 1 | EID, ~ 1 | SID), sigma2=c (0, NA), tdist=TRUE, data= dataset)
> anova (overall, modelnovar2)
```

# between-study variance (significant)

```
> # step 5.2: build a two-level model without between-study variance
> modelnovar3 <- rma.mv (yi, vi, random = list (~ 1 | EID, ~ 1 | SID), sigma2=c (NA, 0), tdist=TRUE, data= dataset)
> anova (overall, modelnovar3)
```

# Determining how the total variance is distributed over the three levels of the meta-analytic model;

```
> # step 5.3: determining how the total variance is distributed over the three levels of the meta-analytic model
> n <- length (dataset $vi)
> list.inverse.variances <- 1 / (dataset $vi)
> sum.inverse.variances <- sum(list.inverse.variances)
> squared.sum.inverse.variances <- (sum.inverse.variances) ^ 2
> list.inverse.variances.square <- 1 / (dataset $vi^2)
> sum.inverse.variances.square <-  sum(list.inverse.variances.square)
> numerator <- (n - 1) * sum.inverse.variances
> denominator <- squared.sum.inverse.variances - sum.inverse.variances.square
> estimated.sampling.variance <- numerator / denominator
> I2_1 <- (estimated.sampling.variance) / (overall$sigma2[1] + overall$sigma2[2] + estimated.sampling.variance)
> I2_2 <- (overall$sigma2[1]) / (overall$sigma2[1] + overall$sigma2[2] + estimated.sampling.variance)
> I2_3 <- (overall$sigma2[2]) / (overall$sigma2[1] + overall$sigma2[2] + estimated.sampling.variance)
> amountvariancelevel1 <- I2_1 * 100
> amountvariancelevel2 <- I2_2 * 100
> amountvariancelevel3 <- I2_3 * 100
> #Variance of the effect size see results section
> #Variance at the sampling level
> amountvariancelevel1
> #Variance at the within-study level
> amountvariancelevel2
> #Variance at the between-study level
> amountvariancelevel3
```

funnel plot

```
> # step 6: Test publication bias ####
> # step 6.1: funnel plot
> funnel(overall)
```

Egger's test and Trim and fill for multilevel meta-analysis

```
> # step 6.2: Egger MLMA (Egger's test for multilevel meta-analysis)
> SD  <-sqrt (dataset$vi)
> SE  <-SD/sqrt(dataset$N)
> sei <-SE
> ##simply by including the standard error of the effect size (or a related measure of precision, such as the sample size) as a moderator. Say that data includes a variable called sei for the standard error of each effect size.
> egger_multi <- rma.mv (yi = yi, V = sei^2,random = list(~ 1 | EID, ~ 1 | SID), mods = ~ sei, data = dataset)
> summary(egger_multi)
```

```
> # step 6.3: Trim and fill for multilevel meta-analysis
> h <- rma.mv(yi, vi, random= list(~ 1 | EID, ~ 1 | SID), data= dataset)
> h <- rma.mv(yi, vi, random= ~ 1 | EID/SID, data= dataset)
> pooled_d<-h$b[1]
> d<-dataset$yi
> R0_func<-function(d, pooled_d){
+   d_difference1=0
+   rank_positive=0
+   final_rank=0
+   d_difference<- d-pooled_d
+   for(i in 1:length(d_difference)){
+     d_difference1[i]<- if ( d_difference[i]<0) d_difference[i]*-1
+     else d_difference[i] }
+   rank_positive<-rank(d_difference1)
+   for(i in 1:length(d_difference)){
+     final_rank[i]<- if (d_difference[i]<0) rank_positive[i]*-1
+     else rank_positive[i]}
+   O=length(d_difference)-(min(final_rank)*-1)
+   R0_ps= O-1
+   R0=if (R0_ps<0) 0 else  R0_ps
+   print(R0)
+ }
> L0_func<-function(d, pooled_d){
+   d_difference1=0
+   rank_positive=0
+   d_difference<- d-pooled_d
+   d_difference1=0
+   only_positive=0
+   final_rank=0
+   N_ES<-length(d)
+   for(i in 1:length(d_difference)){
+     d_difference1[i]<- if ( d_difference[i]<0) d_difference[i]*-1
+     else d_difference[i] }
+   rank_positive<-rank(d_difference1)
+   for(i in 1:length(d_difference)){
+     final_rank[i]<- if (d_difference[i]<0) rank_positive[i]*-1
+     else rank_positive[i]
+     only_positive[i]<- if (final_rank[i]<0) 0
+     else final_rank[i]}
+   t=sum(only_positive)
+   L0_ps=(4*t-N_ES*(N_ES+1))/(2*N_ES-1)
+   L0= if (L0_ps<0) 0 else  L0_ps
+   print(L0)
+ }
> R0_func(d, pooled_d)
> L0_func(d, pooled_d)
```

#Sensitivity Analyses

##leave-one-out analysis

```
> # step 7: Sensitivity Analyses ####
> #leave-one-out analysis for multilevel meta-analysis
> k=nrow(dataset)
> k

> leave1out.estimate <- vector(length = k)
> leave1out.estimate

> for (i in 1:k) {
+   res2 <- rma.mv(yi=yi,
+                V=vi,
+                random = ~ 1 | SID/EID,
+                tdist = TRUE,
+                data = dataset [-i,],
+                method = "REML")
+   leave1out.estimate[i] <-res2$b[1]
+ }
> leave1out.estimate

> range (leave1out.estimate)
```

moderator analysis

```
> # step 8: Testing moderators: Sample type ####
> # step 8: testing categorical moderators: (dummy1= Employee, dummy2= Student)
> Employee <- rma.mv (yi, vi, mods = ~ Student, random = list (~ 1 | EID, ~1 | SID), tdist = TRUE, data=dataset)

> summary (Employee, digits=3)

> Student <- rma.mv (yi, vi, mods = ~ Employee, random = list (~ 1 | EID, ~1 | SID), tdist = TRUE, data=dataset)

> summary (Student, digits=3)

> # step 9: Testing moderators: Personality measurement types ####
> # step 9: testing categorical moderators: (dummy1= NEO, dummy2= IPIP， dummy3= TIPI, dummy4= BFI, dummy5= O
thers)
> NEO <- rma.mv (yi, vi, mods = ~ IPIP + TIPI + BFI + Others, random = list (~ 1 | EID, ~1 | SID), tdist = TRUE, data= da
taset)

> summary (NEO)

> IPIP <- rma.mv (yi, vi, mods = ~ NEO + TIPI + BFI + Others, random = list (~ 1 | EID, ~1 | SID), tdist = TRUE, data= da
taset)
> summary (IPIP)
```

```
> TIPI <- rma.mv (yi, vi, mods = ~ NEO + IPIP + BFI + Others, random = list (~ 1 | EID, ~1 | SID), tdist = TRUE, data= da
taset)

> summary (TIPI)

> BFI <- rma.mv (yi, vi, mods = ~ NEO + IPIP + TIPI + Others, random = list (~ 1 | EID, ~1 | SID), tdist = TRUE, data= da
taset)

> summary (BFI)

> Others <- rma.mv (yi, vi, mods = ~ NEO + IPIP + TIPI + BFI, random = list (~ 1 | EID, ~1 | SID), tdist = TRUE, data= da
taset)

> summary (Others)

> # step 10: Testing moderators: Social individualism index (cultural background index)##
> # continuous variable
> #Centering on continuous variables(c = centralized mean)
> ## Social individualism indexc
> Social individualism indexc <- scale(dataset$Social individualism index (cultural background index), center = TRUE , sca
le = FALSE )[,]
> Social individualism indexc

> cultural background <- rma.mv(yi, vi, mods = ~ Social individualism indexc, random = list(~ 1 | EID, ~ 1 | SID),
+                   tdist=TRUE, data=dataset)

> summary(cultural background, digits=3)

> # continuous variable
> #Centering on continuous variables(c = centralized mean)
> Gender (female ratio)c <- scale(dataset$Gender (female ratio), center = TRUE , scale = FALSE )[,]
> Gender (female ratio)c

> Gender <- rma.mv(yi, vi, mods = ~ Gender (female ratio)c, random = list(~ 1 | EID, ~ 1 | SID),
+                   tdist=TRUE, data=dataset)

> summary(Gender, digits=3)
```
